# Supplementary material for: ER assembly of SNARE complexes mediating formation of partitioning membrane in Arabidopsis cytokinesis
Source: eLife. 2017 May 19;6:e25327. doi: 10.7554/eLife.25327 (PMC5438246; doi:10.7554/eLife.25327)
Supplement: Supplementary file 1. — (b) Primers used in this study DOI: http://dx.doi.org/10.7554/eLife.25327.010 [file elife-25327-supp1.doc]

**Supplementary File**

**Supplementary file 1a. Frequency of cytokinetic cells in mutant seedling roots.**

| Genotype | -BFA (mean ± SD)* | +BFA (mean ± SD)* |
| --- | --- | --- |
| WT (Col) | 35±6 | 35±9 |
| *big3* | 38±6 | 40±6 |
| *gnl1 GNL1^BFA-sens.^* | 32±10 | 32±9 |

* The mean value of cytokinetic cells per seedling root was determined by counting the anti-α-tubulin-stained phragmoplasts in the entire division zone (see supplementary Figure 1 for immunofluorescence images). -BFA, mock treatment; +BFA, BFA treatment; SD, standard deviation based on the number of seedling roots (n=7). The measurements were technically repeated twice.

**Supplementary file 1b. Primers used in this study**

| **Primers** | **Sequences (5’-3’)** |
| --- | --- |
| PEP12-XbaI-5 | AAAAAATCTAGAATGAGTTTCCAAGATCTC |
| PEP12-EcoRI-3 | TTTTTTGAATTCTTAGACCAAGACAACGAT |
| GFP-AttB1-5 | AAAAAGCAGGCTGTATGAGTAAAGGAGAAGAACTTTTCAC |
| SNAP33-AttB2-3 | AGAAAGCTGGGTCCTATTTGCCCAACAAACGTCGGCCTCGTTGGTTTGATTG |
| YFP-AttB1-5 | AAAAAGCAGGCTATATGGTGAGCAAGGGCG |
| NPSN11-AttB2-3 | AGAAAGCTGGGTCTCAGTAATGGTTCCAGAGCAGAC |
